# Non-Equilibrium Entropy and Irreversibility in Generalized Stochastic Loewner Evolution from an Information-Theoretic Perspective

**DOI:** 10.3390/e23091098

**Published:** 2021-08-24

**Authors:** Yusuke Shibasaki, Minoru Saito

**Affiliations:** Department of Correlative Study in Physics and Chemistry, Graduate School of Integrated Basic Sciences, Nihon University, Setagaya, Tokyo 156-8550, Japan; yshib@chs.nihon-u.ac.jp

**Keywords:** stochastic Loewner evolution (SLE), entropy production, non-equilibrium statistical mechanics, Langevin equation, Fokker-Planck equation, time irreversibility, information theory

## Abstract

In this study, we theoretically investigated a generalized stochastic Loewner evolution (SLE) driven by reversible Langevin dynamics in the context of non-equilibrium statistical mechanics. Using the ability of Loewner evolution, which enables encoding of non-equilibrium systems into equilibrium systems, we formulated the encoding mechanism of the SLE by Gibbs entropy-based information-theoretic approaches to discuss its advantages as a means to better describe non-equilibrium systems. After deriving entropy production and flux for the 2D trajectories of the generalized SLE curves, we reformulated the system’s entropic properties in terms of the Kullback–Leibler (KL) divergence. We demonstrate that this operation leads to alternative expressions of the Jarzynski equality and the second law of thermodynamics, which are consistent with the previously suggested theory of information thermodynamics. The irreversibility of the 2D trajectories is similarly discussed by decomposing the entropy into additive and non-additive parts. We numerically verified the non-equilibrium property of our model by simulating the long-time behavior of the entropic measure suggested by our formulation, referred to as the relative Loewner entropy.

## 1. Introduction

Although the irreversibility of non-equilibrium systems has been discussed in numerous fields for decades, the difficulties accompanying their theoretical formulation essentially involve the definition of the concept of *entropy* [1,2,3]. Since the pioneering study by Prigogine et al. [4], entropy production describing the dissipative open systems far from equilibrium has been studied by employing Gibbs entropy-based approaches [5,6,7,8,9,10,11,12,13,14]. These formulations assume that time irreversibility in non-equilibrium states is characterized by a non-zero-entropy production rate of the system, and time reversibility (or time symmetry) holds only when the system is in an equilibrium state with a zero-entropy production rate [7,8,9]. The validity of the assumptions has been proven by various types of the fluctuation theorem (FT) [15,16,17] combined with the stochastic dynamics described by Langevin and Fokker–Planck equations [8,9,18,19,20], etc. One of the advantages of the Gibbs entropy-based approach is that it is compatible with the Shannonian *information entropy*. Whereas the information entropy was originally a measure of uncertainty of the events consistently used for describing equilibrium systems, the concept of *information* is often adopted into the theory of thermodynamics as a quantity we obtain by the measurement of the system [2,21,22]. Due to its utility, the Gibbs–Shannon entropy-based approaches (i.e., information-theoretic perspectives) for non-equilibrium systems have also been developed based on several different methodologies [23,24,25,26,27]. In particular, the recent advance of this perspective has enabled investigation of the generic properties of the entropy production rate [25,26], and the related results were found to be applicable to specific physical problems (e.g., heat conduction [27]).

However, even with the above-mentioned approaches, the characterization of non-equilibrium systems using quantities from equilibrium physics still includes several controversial issues (e.g., the measure describing non-equilibrium stationary states). Notably, as an attempt to overcome this difficulty, an alternative form of the Gibbs–Shannon entropy was proposed in a different context, based on the non-additivity of the ensembles of non-equilibrium states [28,29]. These problems concerning *non-equilibrium entropy* can be reduced to the estrangement between non-equilibrium physics and well-established equilibrium physics.

A previous study by the authors indicated that the stochastic Loewner evolution (SLE) proposed by Schramm [30,31] provides a bridge between the equilibrium and non-equilibrium (i.e., reversible and irreversible) statistical mechanics systems [32]. The SLE theory typically describes the conformally invariant geometries (curves) in various two-dimensional (2D) statistical mechanics models, whose time evolutions are determined by the Loewner equation [33] driven by Brownian motion (Wiener process). In ref. [32], the authors reported that the framework of the SLE can be regarded as a system that encodes the (microscopically) irreversible trajectories of the curves into the reversible driving functions. This shows, in other words, that the 2D non-equilibrium trajectories described by the SLE are the images of the equilibrium systems under the conformal transformations uniquely determined by the Loewner equation.

In this study, we developed this perspective by using a generalized SLE framework, employing a driving function governed by the Langevin equation (described in Section 2). We present an information-theoretic perspective for the thermodynamics of the SLE to show the advantages of encoding non-equilibrium systems into equilibrium systems. Motivated by the above, our aim was to formulate the Gibbs–Shannon entropy-based relations between curves in the physical plane and driving functions in the mathematical plane in a generalized SLE framework (presented in Section 3). The main tools of the first step of our analysis are the Langevin and Fokker–Planck equations describing the trajectories of the tip of the curve, which are available only when the corresponding driving function satisfies the time symmetric property [31,34,35,36,37]. After deriving several basic non-equilibrium entropic relations (e.g., entropy production and flux, Jarzynski equality [38]), we deduce these relations in terms of the Kullback–Leibler (KL) divergence [39,40,41,42] to introduce an extended second law of the thermodynamics. Subsequently, by considering the phase space deformation induced by the conformal maps determined by the Loewner equation, we suggest a novel irreversibility measure, which we call the *relative Loewner entropy*. We also remark that the relative Loewner entropy, defined as a probabilistic divergence between the trajectory of the curve and the driving function, is closely related to the Lyapunov-type exponent of the conformal map in the Loewner equation. Using these quantities, numerical simulations were performed to verify non-equilibrium states of the generalized SLE curves (in Section 4). In the discussion (Section 5), we reinterpret the statistical physical meanings of our results, most of which rely on information theory, in relation to the problem of the determination of a non-equilibrium entropy.

## 2. Model

### 2.1. Chordal Loewner Evolution 

We consider the chordal Loewner evolution described as follows. Let $\gamma_{\left[0,\;t\right]}$ be a simple curve parametrized by time t on the upper half complex plane ℍ, starting from the origin. The following Loewner equation yields a family of time-dependent conformal maps $g_{t}$ from $ℍ\setminus \gamma_{\left[0,\;t\right]}$ to ℍ:

(1) $$\frac{\partial g_{t}\left(z\right)}{\partial t}=\frac{2}{g_{t}\left(z\right)-\left(\xi_{t}-\xi_{0}\right)},\;\;\;\;\;g_{0}\left(z\right)=z,\;\;\;\;\;z\in ℍ,$$

where $\xi_{t}$ is a one-dimensional real-valued time function called the driving function [30,31,33] defined in the following subsection. $\xi_{0}$ is the initial condition of the driving function. When $\xi_{t}$ corresponds to the Brownian motion (i.e., Wiener process), Equation (1) describes the SLE process in a usual sense. The conformal map satisfying the Loewner equation in Equation (1) is given as follows [33]:

(2) $$g_{t}\left(z\right)=z+\frac{2t}{z}+O\left(\left|z^{-2}\right|\right),\;\;\;\;\;\mathrm{as}\;\;\;z\to \infty \;.$$

The relation between the tip of the curve $\gamma_{t}$ and the driving function $\xi_{t}$ is expressed as follows [33]:

(3) $$\underset{z\to \gamma_{t}}{\lim}g_{t}\left(z\right)=\xi_{t}-\xi_{0}.$$

Therefore, from Equations (1) and (3), it is evident that the family of $g_{t}$ encodes the history of the time evolution of the tip $\gamma_{\left[0,\;t\right]}$ into the driving function $\xi_{\left[0,\;t\right]}$ Notably, this transformation has a one-to-one correspondence between the curves and driving functions, and is reversible in the sense of the uniqueness of the inverse transformation, i.e., $\;\underset{w\to \xi_{t}-\xi_{0}}{\lim}g_{t}{}^{-1}\left(w\right)=\gamma_{t}\;,\;\left(w\in ℍ\right)$. However, in practice, the exact determination of $g_{t}$ is difficult and specific algorithms are required. Indeed, this encoding mechanism is a physically non-trivial and meaningful process, as we show later. In the following, we refer to the upper half-plane where the driving function evolves on the real axis as the *mathematical plane*, and that where the curve evolves as the *physical plane*.

### 2.2. Langevin Dynamics as a Driving Function

We consider that the driving function $\xi_{t}$ of the Loewner evolution is governed by the following Langevin equation [43]:

(4) $$\frac{d\xi_{t}}{dt}=\alpha \left(\xi_{t}\right)+\sqrt{\kappa }\frac{dB_{t}}{dt},$$

where $B_{t}$ is the standard Brownian motion and κ is a diffusion parameter. Note that κ is a main factor to determine the fractal dimension of the curve in the physical plane. The drift term $\alpha \left(\xi_{t}\right)$ is assumed to be a conservative force that has a potential function $V\left(\xi ,t\right)$ satisfying:

(5) $$\alpha \left(\xi ,t\right)=-\frac{\partial V\left(\xi ,t\right)}{\partial \xi }.$$

This condition guarantees that $\xi_{t}$ can be a time-reversible process [44]. The associated Fokker–Planck equation is described as [7]:

(6) $$\frac{\partial p\left(\xi ,t\right)}{\partial t}=\left[\frac{\kappa }{2}\frac{\partial^{2}}{\partial \xi^{2}}-\frac{\partial }{\partial \xi }\alpha \left(\xi \right)\right]p\left(\xi ,t\right).$$

Here, $p\left(\xi ,t\right)=\langle \delta \left(\xi -\xi_{t}\right)\rangle$, where the brackets denote the ensemble average. For convenience, we define the probability current as:

(7) $$J\left(\xi ,t\right)=-\left[\frac{\kappa }{2}\frac{\partial }{\partial \xi }-\alpha \left(\xi \right)\right]p\left(\xi ,t\right).$$

Then, the Fokker–Planck equation in Equation (6) is expressed as:

(8) $$\frac{\partial p\left(\xi ,t\right)}{\partial t}=-\frac{\partial }{\partial \xi }J\left(\xi ,t\right).$$

We assume the scenario where $\xi_{t}$ is in the equilibrium state is characterized by:

(9) pξ,t=psξt=1Ze−2Vξtκ.

Here, Z is a normalization constant, and $p_{s}\left(\xi_{t}\right)$ is a stationary probability distribution that satisfies $J\left(\xi ,t\right)=0$ [7,8,9]. In addition, we assume that $V\left(\xi ,t\right)$ is symmetric in ξ such that the driving function satisfies the relation that $\xi_{t}$ and $-\xi_{t}$ have the same probability distribution.

## 3. General Formulation

### 3.1. Equilibrium Condition on Mathematical Plane

In the following formulations, we impose the equilibrium condition on the driving function in the mathematical plane from the initial condition, which is characterized by the zero-entropy production rate of $\xi_{t}$ constructed above. Let us define the Gibbs entropy of $\xi_{t}$ as follows:

(10) $$S^{m}=-\int p\left(\xi ,t\right)\ln p\left(\xi ,t\right)d\xi =\langle s^{m}\rangle {}_{\xi_{t}},$$

where $s^{m}=-\ln p\left(\xi ,t\right)\left[=-\ln p_{s}\left(\xi_{t}\right)\right]$ and $\langle \;\cdot \;\rangle_{\xi_{t}}$ denotes the ensemble average over all realizations of the driving function. Here, the Boltzmann constant $k_{B}$ is regarded as 1. $S^{m}>0$ and $dS^{m}/dt=0$ can be derived from Equations (9) and (10), indicating the non-negative and time-independent properties of the equilibrium entropy.

Furthermore, we assume the following detailed balance condition for the driving function [43]:

(11) $$p\left(\xi_{n}|\xi_{n-1}\right)p\left(\xi_{n-1}|\xi_{n-2}\right)\cdots p\left(\xi_{1}|\xi_{0}\right)p_{s}\left(\xi_{0}\right)=p\left(\xi_{0}|\xi_{1}\right)p\left(\xi_{1}|\xi_{2}\right)\cdots p\left(\xi_{n-1}|\xi_{n}\right)p_{s}\left(\xi_{n}\right).$$

Here, $p\left(A|B\right)$ is the transition probability from state B to A and $n\;\left(\geq 1\right)$ is the integer index satisfying t=nτ, where τ is a sufficiently small-time interval. Let us define:

(12) $$P\left[\xi_{path}\left(n\right)\right]=p\left(\xi_{n}|\xi_{n-1}\right)p\left(\xi_{n-1}|\xi_{n-2}\right)\cdots p\left(\xi_{1}|\xi_{0}\right)p_{s}\left(\xi_{0}\right),$$

and:

(13) $$\tilde{P}\left[{\tilde{\xi }}_{path}\left(n\right)\right]=p\left(\xi_{0}|\xi_{1}\right)p\left(\xi_{1}|\xi_{2}\right)\cdots p\left(\xi_{n-1}|\xi_{n}\right)p_{s}\left(\xi_{n}\right).$$

Then, we define the ratio between these probabilities as $R_{m}\equiv$ $P\left[\xi_{path}\left(n\right)\right]/\tilde{P}\left[{\tilde{\xi }}_{path}\left(n\right)\right]$, so that:

(14) $$\ln R_{m}=\ln\frac{P\left[\xi_{path}\left(n\right)\right]}{\tilde{P}\left[{\tilde{\xi }}_{path}\left(n\right)\right]}=0,$$

which follows from Equation (11). From the formulation using the master equations [7,8,9,10,45], $\ln R_{m}=0$ suggests that there is no entropy production inside the system for each trajectory and the microscopic time reversibility is guaranteed for all time.

### 3.2. Entropy Production in Physical Plane

We investigate the entropy production of the trajectory of the curve in the physical plane. We demonstrate the irreversible and dissipative character of the SLE curve, which differs from that of the driving function. We mainly use the Langevin and Fokker–Planck equations for SLE curve, which are available due to the detailed balanced condition. The formulation using the backward Loewner evolution [31,34,35,36,37] shows that if the driving function is time-symmetric (i.e., $-\xi_{t}$ and $\xi_{-t}$ have the same probability distribution) and has stationary increments, the probability distribution for the time evolution of the tip of the curve $z_{t}$ is the same of that of $\left(x_{t},\;y_{t}\right)$, described by the following two-dimensional Langevin equation:

(15) $$\frac{dx_{t}}{dt}=-\frac{2x_{t}}{x_{t}{}^{2}+y_{t}{}^{2}}-\alpha \left(\xi_{t}\right)-\sqrt{\kappa }\frac{dB_{t}}{dt}\;\frac{dy_{t}}{dt}=\frac{2y_{t}}{x_{t}{}^{2}+y_{t}{}^{2}}.\;\;\;\;\;\;\;$$

Here, we adopt the initial condition of $x_{0}=0$ and $y_{0}=\varepsilon$, where ε is an infinitesimal positive constant [35]. In the limit of $x_{t}\to \pm \infty$ and $y_{t}\to \infty$, we obtain $dx_{t}/dt\to -\alpha \left(\xi_{t}\right)-\sqrt{\kappa }dB_{t}/dt$ and $dy_{t}/dt\to 0$. We note that this condition is an equilibrium state of the curve trajectory characterized by the probability that is the same as that of the driving function. The Fokker–Planck equation associated with Equation (15) is expressed as follows:

(16) $$\frac{\partial p\left(x,y,t\right)}{\partial t}=\left\{\frac{\kappa }{2}\frac{\partial^{2}}{\partial x^{2}}+\frac{\partial }{\partial x}\left[\frac{2x}{x^{2}+y^{2}}+\alpha \left(\xi \right)\right]-\frac{\partial }{\partial y}\frac{2y}{x^{2}+y^{2}}\right\}p\left(x,y,t\right).$$

Here, $p\left(x,y,t\right)=\langle \delta \left(x-x_{t}\right)\delta \left(y-y_{t}\right)\rangle$, where the ensemble average is calculated over all realizations of the curves. For the latter formulations, we take:

(17) $$\chi \left(x,\;y,\;t\right)=\frac{\kappa }{2}\frac{\partial }{\partial x}p\left(x,\;y,\;t\right)+\left[\frac{2x}{x^{2}+y^{2}}+\alpha \left(\xi \right)\right]p\left(x,\;y,\;t\right)$$

and:

(18) $$\psi \left(x,\;y,\;t\right)=\frac{2y}{x^{2}+y^{2}}p\left(x,\;y,\;t\right).$$

Substituting Equations (17) and (18) into Equation (16), the Fokker–Planck equation for the trajectory of curve is expressed as:

(19) $$\frac{\partial p\left(x,y,t\right)}{\partial t}=\frac{\partial }{\partial x}\chi \left(x,\;y,\;t\right)-\frac{\partial }{\partial y}\psi \left(x,\;y,\;t\right).$$

Subsequently, we define the time-dependent Gibbs entropy for the trajectory of the curve as $S^{p}\left(t\right)$, which is expressed as:

(20) $$S^{p}\left(t\right)=-\iint p\left(x,y,t\right)\ln p\left(x,y,t\right)dxdy=\langle s^{p}\rangle {}_{x_{t},y_{t}},$$

where $s^{p}=-\ln p\left(x,\;y,t\right)$, and $\langle \;\cdot \;\rangle_{x_{t},y_{t}}$ denotes the ensemble average over all possible realizations of $\left(x_{t},\;y_{t}\right)$. We are interested in the changing rate of $S^{p}\left(t\right)$, which was formulated by Prigogine et al. as the following [4,7,8,32]:

(21) $$\frac{dS^{p}\left(t\right)}{dt}=\frac{dS_{i}^{p}\left(t\right)}{dt}-\frac{dS_{e}^{p}\left(t\right)}{dt},$$

where $dS_{i}^{p}\left(t\right)/dt$ is the entropy production rate inside the system, which is non-negative because of the second law of thermodynamics. The second term on the right-hand side of Equation (21), $dS_{e}^{p}\left(t\right)/dt$, is the entropy flux rate from the system to the external environment. If the system is stationary, $dS_{i}^{p}\left(t\right)/dt=dS_{e}^{p}\left(t\right)/dt$, whereas if the system is in equilibrium, $dS_{i}^{p}\left(t\right)/dt=dS_{e}^{p}\left(t\right)/dt=$ 0. In both scenarios, $S^{p}\left(t\right)$ assumes a constant value, otherwise $S^{p}\left(t\right)$ changes depending on the time and the system is in non-equilibrium [7,8]. For subsequent discussions, we define the entropy production $s_{i}^{p}$ and entropy flux $s_{e}^{p}$ for the individual trajectories of the tips of the curves as those satisfying $dS_{i}^{p}\left(t\right)/dt=\langle ds_{i}^{p}/dt\rangle_{x_{t},y_{t}}$ and $dS_{e}^{p}\left(t\right)/dt=\langle ds_{e}^{p}/dt\rangle_{x_{t},y_{t}}$.

Hereafter, we apply the entropic formulation in Equation (21) to the SLE curve in our model, using Equations (17)–(20), and performing partial integrations. The time derivative of $S^{p}\left(t\right)$ can be calculated as [32]:

(22) $$\frac{dS^{p}}{dt}=\iint \frac{2}{\kappa }\frac{{\left[\chi \left(x,y,t\right)\right]}^{2}}{p\left(x,y,t\right)}dxdy-\iint \frac{2}{\kappa }\left(\frac{2x}{x^{2}+y^{2}}+\alpha \left(\xi \right)\right)\chi \left(x,y,t\right)dxdy-\iint \frac{2y}{x^{2}+y^{2}}\frac{\partial }{\partial y}p\left(x,y,t\right)dxdy.$$

Here, we dropped the boundary terms whose $p\left(x,y,t\right)$ tends to zero when x→±∞ or y→±∞. Because the first term of the right-hand side of Equation (22) is non-negative, we can identify it with the entropy production rate, that is:

(23) $$\frac{dS_{i}^{p}\left(t\right)}{dt}=\iint \frac{2}{\kappa }\frac{{\left[\chi \left(x,y,t\right)\right]}^{2}}{p\left(x,y,t\right)}dxdy\geq 0.$$

The equality holds when $\chi \left(x,\;y,\;t\right)=0$, and this is a necessary condition for thermal equilibrium. In this framework, the entropy production rate is described in terms of the free energy F of the system as $dS_{i}^{p}\left(t\right)/dt=-dF/dt$ [13]. Therefore, by combination with Equation (23), dF/dt≤0 can be derived. This outcome is interpreted as the *H*-theorem for the trajectory of the SLE curve, and it ensures that the system is thermodynamically irreversible in time, except for the equilibrium condition [46].

In addition, the second and third terms of the right-hand side of Equation (22) are interpreted as the contributions for the entropy flux rate. Performing the partial integration and using the definition of the ensemble average, the entropy flux rate is expressed as [32]:

(24) $$\frac{dS_{e}^{p}\left(t\right)}{dt}={\langle \frac{2}{\kappa }{\left(\frac{2x_{t}}{x_{t}{}^{2}+y_{t}{}^{2}}+\alpha \left(\xi_{t}\right)\right)}^{2}-\frac{\partial \alpha \left(\xi_{t}\right)}{\partial x}\rangle }_{x_{t},y_{t}}.$$

Then, the entropy flux (from t=0) for individual trajectories, $s_{e}^{p}$, is calculated by the following integral:

(25) $$s_{e}^{p}=\overset{t}{\underset{0}{\int }}\left[\frac{2}{\kappa }{\left(\frac{2x_{t}}{x_{t}{}^{2}+y_{t}{}^{2}}+\alpha \left(\xi_{t}\right)\right)}^{2}-\frac{\partial \alpha \left(\xi_{t}\right)}{\partial x}\right]dt,$$

which is the total amount of entropy dissipated to the external environment for each trajectory of the curves. Note that $(x_{t},\;y_{t}$) and Rezt, Im(zt) have the same joint probability distribution. The increase in $s_{e}^{p}$ indicates that the generalized SLE curve remains in a non-equilibrium state, contrary to the fact that the corresponding driving function is in an equilibrium state from the initial conditions.

### 3.3. Jarzynski Equality for Generalized SLE Curve

We derive the equality governing the time-irreversible trajectories of the SLE curve, which was originally derived by Jarzynski [38] and applied to stochastic trajectories by Seifert [10]. Let us denote the discretized points on curve $\gamma_{\left[0,\;t\right]}$ as $\gamma_{\left[0,\;n\right]}=\left\{z_{0}\left(=0\right),z_{1},z_{2},\ldots ,z_{n}\right\}$. Then, in the same manner as Equations (12) and (13), we define:

(26) $$P\left[z_{path}\left(n\right)\right]=p\left(z_{n}|z_{n-1}\right)p\left(z_{n-1}|z_{n-2}\right)\cdots p\left(z_{1}|z_{0}\right)p\left(z_{0}\right)$$

and:

(27) $$\tilde{P}\left[{\tilde{z}}_{path}\left(n\right)\right]=p\left(z_{0}|z_{1}\right)p\left(z_{1}|z_{2}\right)\cdots p\left(z_{n-1}|z_{n}\right)p\left(z_{n}\right).$$

Subsequently, we define the ratio of these probabilities as $R_{p}\equiv P\left[z_{path}\left(n\right)\right]/\tilde{P}\left[{\tilde{z}}_{path}\left(n\right)\right]$. From the formulations using master equations, $\ln R_{p}$ is expressed in terms of the entropy flux as follows [8,10]:

(28) $$\ln R_{p}=\ln\frac{P\left[z_{path}\left(n\right)\right]}{\tilde{P}\left[{\tilde{z}}_{path}\left(n\right)\right]}=s_{e}^{p}+\ln\frac{p\left(z_{0}\right)}{p\left(z_{n}\right)}.$$

Here, the individual entropy flux $s_{e}^{p}$ is given by Equation (25). From Equation (28), Jarzynski equality can be derived as [8,10]:

(29) $$\langle e^{-\ln R_{p}}\rangle_{z_{path}}=\underset{z_{path}}{\sum }P\left[z_{path}\left(n\right)\right]e^{-\ln R_{p}}\;\;\;\;\;\;\;\;\;\;\;\;\;=\underset{{\tilde{z}}_{path}}{\sum }\tilde{P}\left[{\tilde{z}}_{path}\left(n\right)\right]\;=1.\;\;\;\;\;\;\;\;\;\;$$

Here, $\langle \;\cdot \;\rangle_{z_{path}}$ denotes the ensemble average over the forward path of the curve. Using Equations (25) and (28), Equation (29) is expressed as:

(30) $$\langle \exp{\left\{-\overset{t}{\underset{0}{\int }}\left[\frac{2}{\kappa }{\left(\frac{2x_{t}}{x_{t}{}^{2}+y_{t}{}^{2}}+\alpha \left(\xi_{t}\right)\right)}^{2}-\frac{\partial \alpha \left(\xi_{t}\right)}{\partial x}\right]dt+\ln\frac{p\left(z_{n}\right)}{p\left(z_{0}\right)}\right\}\rangle }_{z_{path}}=1.$$

This is the Jarzynski equality for the generalized SLE curve, which is applicable regardless of whether the curve trajectory is in an equilibrium or non-equilibrium state.

### 3.4. KL Divergence Approach

Although we showed the underlying entropic law in the trajectories of generalized SLE curves using previously studied formulations, the non-equilibrium characteristic of the 2D trajectory in the physical plane is generated by the transformation of the reversible driving function. To clarify this encoding property of the SLE in terms of information theory, we take an approach using the KL divergence. In the following formulation, we eliminate the restrictions on initial conditions, assuming that the driving function is in a relaxation process to the equilibrium state, which requires a certain length of time.

Subtracting Equation (28) from Equation (14) yields:

(31) $$\ln(R_{m}/R_{p})=\ln\frac{P\left[\xi_{path}\left(n\right)\right]}{\tilde{P}\left[{\tilde{\xi }}_{path}\left(n\right)\right]}-\ln\frac{P\left[z_{path}\left(n\right)\right]}{\tilde{P}\left[{\tilde{z}}_{path}\left(n\right)\right]}\;\;\;\;\;\;\;\;\;\;\;\;\;\;=\ln\frac{\tilde{P}\left[{\tilde{z}}_{path}\left(n\right)\right]}{\tilde{P}\left[{\tilde{\xi }}_{path}\left(n\right)\right]}-\ln\frac{P\left[z_{path}\left(n\right)\right]}{P\left[\xi_{path}\left(n\right)\right]}.$$

We denote KL divergences between the forward paths of the driving function and the curve as the following:

(32) $$D\left(z_{path}∥\xi_{path}\right)=\underset{z_{path}\left(n\right)}{\sum }P\left[z_{path}\left(n\right)\right]\ln\frac{P\left[z_{path}\left(n\right)\right]}{P\left[\xi_{path}\left(n\right)\right]}.$$

Similarly, for the backward paths we denote:

(33) $$\tilde{D}\left({\tilde{z}}_{path}∥{\tilde{\xi }}_{path}\right)=\underset{{\tilde{z}}_{path}\left(n\right)}{\sum }\tilde{P}\left[{\tilde{z}}_{path}\left(n\right)\right]\ln\frac{\tilde{P}\left[{\tilde{z}}_{path}\left(n\right)\right]}{\tilde{P}\left[{\tilde{\xi }}_{path}\left(n\right)\right]}.$$

Then, we define $d\left(z_{path}∥\xi_{path}\right)\equiv \ln\frac{P\left[z_{path}\left(n\right)\right]}{P\left[\xi_{path}\left(n\right)\right]}$ and $\tilde{d}\left({\tilde{z}}_{path}∥{\tilde{\xi }}_{path}\right)\equiv \ln\frac{\tilde{P}\left[{\tilde{z}}_{path}\left(n\right)\right]}{\tilde{P}\left[{\tilde{\xi }}_{path}\left(n\right)\right]}$, so that:

$$D\left(z_{path}∥\xi_{path}\right)=\langle d{\left(z_{path}∥\xi_{path}\right)\rangle }_{z_{path}},$$

and:

(34) $$\tilde{D}\left({\tilde{z}}_{path}∥{\tilde{\xi }}_{path}\right)=\langle \tilde{d}{\left({\tilde{z}}_{path}∥{\tilde{\xi }}_{path}\right)\rangle }_{{\tilde{z}}_{path}}.$$

Using these expressions, Equation (31) is expressed as:

(35) $$R_{m}/R_{p}=\exp\left[\tilde{d}\left({\tilde{z}}_{path}∥{\tilde{\xi }}_{path}\right)-d\left(z_{path}∥\xi_{path}\right)\right].$$

For the left-hand side of Equation (35), we take the dual ensemble average with respect to $z_{path}$ and ${\tilde{\xi }}_{path}$, hence:

(36) $${\langle \frac{R_{m}}{R_{p}}\rangle }_{z_{path,\;}{\tilde{\xi }}_{path}}=\underset{{\tilde{\xi }}_{path}}{\sum }\underset{z_{path}}{\sum }P\left[z_{path}\left(n\right)\right]\tilde{P}\left[{\tilde{\xi }}_{path}\left(n\right)\right]e^{\ln\left(R_{m}/R_{p}\right)}\;\;\;=\underset{\xi_{path}}{\sum }\underset{{\tilde{z}}_{path}}{\sum }\tilde{P}\left[{\tilde{z}}_{path}\left(n\right)\right]P\left[\xi_{path}\left(n\right)\right]\;\;\;\;\;\;\;=1.\;\;\;\;\;\;\;\;\;\;\;\;\;\;\;\;$$

Because we obtain the relation $\langle R_{m}/R_{p}\rangle_{z_{path,\;}{\tilde{\xi }}_{path}}=1$, using Equation (36), Equation (35) yields:

(37) $$\langle \exp{\left[\tilde{d}\left({\tilde{z}}_{path}∥{\tilde{\xi }}_{path}\right)-d\left(z_{path}∥\xi_{path}\right)\right]\rangle }_{z_{path,\;}{\tilde{\xi }}_{path}}=1.$$

This is an alternative of the Jarzynski equality, which is applicable for the generalized SLE that we defined previously. From the Jensen inequality, the following relation is derived:

(38) $$\langle \exp{\left[\tilde{d}\left({\tilde{z}}_{path}∥{\tilde{\xi }}_{path}\right)-d\left(z_{path}∥\xi_{path}\right)\right]\rangle }_{z_{path,\;}{\tilde{\xi }}_{path}}\geq \exp\left[\langle \tilde{d}{\left({\tilde{z}}_{path}∥{\tilde{\xi }}_{path}\right)\rangle }_{z_{path,\;}{\tilde{\xi }}_{path}}-\langle d{\left(z_{path}∥\xi_{path}\right)\rangle }_{z_{path},\;{\tilde{\xi }}_{path}}\right].$$

Substituting Equation (37) into Inequation (38), we obtain the following inequality:

(39) $$\langle D{\left(z_{path}∥\xi_{path}\right)\rangle }_{\;{\tilde{\xi }}_{path}}\geq \langle d\left(z_{path}∥\xi_{path}\right)-\ln R_{p}+\ln R_{m}\rangle {}_{z_{path,\;}{\tilde{\xi }}_{path}}$$

Using Equation (28), Inequation (39) is transformed as follows:

(40) $$0\leq \langle s_{e}^{p}+\ln\frac{p\left(z_{0}\right)}{p\left(z_{n}\right)}-\ln R_{m}\rangle {}_{z_{path,\;}{\tilde{\xi }}_{path}}.$$

Using the relation $s_{i}^{p}=s_{e}^{p}+\ln\frac{p\left(z_{0}\right)}{p\left(z_{n}\right)}$, the following inequality is derived:

(41) $$0\leq \langle s_{i}^{p}-\ln R_{m}\rangle {}_{z_{path,\;}{\tilde{\xi }}_{path}}$$

This relation can be interpreted as an extension of the second law of thermodynamics. In Inequation (41), the equality holds true if the trajectory is in an equilibrium state characterized by $s_{i}^{p}=\ln R_{m}=0$. Note that $s_{e}^{p}$ in Equation (40) is expressed by Equation (25) only when the time reversibility of the driving function is guaranteed. Considering the above derivation process, the generalized Jarzynski equality in Equation (37) and second law-type relation in Inequations (40) and (41) are generic relations in the sense that they hold true for arbitrary 2D trajectories regardless of the entropic characteristics of corresponding driving functions.

### 3.5. Relative Loewner Entropy

We showed that the non-equilibrium states of the trajectory of the SLE curves are formulated by the Shannon entropy-based KL divergence. Furthermore, we demonstrated the encoding property of the Loewner evolution by characterizing the entropy production and flux in terms of the path probabilities of the curves and the driving functions. In this subsection, we elucidate the intrinsic mechanism that affords this encoding by examining the phase space deformation induced by the conformal map $g_{t}$, which is shown to be a fundamental factor of the irreversibility. We further incorporate a viewpoint of the non-additivity of the entropy, which is a basic concept of non-extensive statistical mechanics [28,29]. In the following, we assume that the driving function is in an equilibrium state.

The relation between the probability for z and w under the conformal map $z=g_{t}^{-1}\left(w\right)$ is expressed as [47,48]:

(42) $$p\left(z\right)=p\left(w\right)\;{\left|\frac{dg_{t}\left(z\right)}{dz}\right|}^{2}.$$

Using the relation $\xi_{t}-\xi_{0}=\underset{z\to \gamma_{t}}{\lim}g_{t}\left(z\right)$, where $\gamma_{t}=z_{t}$ and $p\left(\xi_{t}\right)=p\left(\xi_{t}-\xi_{0}\right)$, in Equation (3) and taking the logarithms, the entropy $s^{p}\left(z_{t}\right)$ for the tip on the curve is described as:

(43) $$s^{p}\left(z_{t}\right)=s^{m}\left(\xi_{t}\right)-\ln{\left|\frac{dg_{t}\left(z_{t}\right)}{dz}\right|}^{2},\;$$

where from Equation (2) for large $z_{t}$:

(44) $$\left|\frac{dg_{t}\left(z_{t}\right)}{dz}\right|=\left|1-\frac{2t}{z_{t}{}^{2}}+O\left(\left|z_{t}{}^{-3}\right|\right)\right|.$$

Here, the entropy $s^{p}\left(z_{t}\right)$ is decomposed into an equilibrium entropy $s^{m}\left(\xi_{t}\right)$ and the rest of the part $-\ln{\left|\frac{dg_{t}\left(z_{t}\right)}{dz}\right|}^{2}$. Because $s^{m}\left(\xi_{t}\right)$ is associated with the time-independent canonical distribution $p_{s}\left(\xi_{t}\right)$ described by Equation (9), it can be referred to as the *additive* part. Contrarily, $\ln{\left|\frac{dg_{t}\left(z_{t}\right)}{dz}\right|}^{2}$ is time dependent as we numerically show later, and referred to as the *non-additive* part in the sense of the non-extensive statistical mechanics [28,29,49]. Notably, the behavior of the non-additive part characterizes non-equilibrium (irreversible) properties of the generalized SLE curve. Let us define $d\left(z_{t}∥\xi_{t}\right)\equiv \ln\frac{p\left(z_{t}\right)}{p\left(\xi_{t}\right)}=\ln{\left|\frac{dg_{t}\left(z_{t}\right)}{dz}\right|}^{2}$. From Equation (43), the time derivative of $s^{p}\left(z_{t}\right)$ is expressed as:

(45) $$\frac{ds^{p}\left(z_{t}\right)}{dt}=-\frac{d}{dt}d\left(z_{t}∥\xi_{t}\right).$$

Here, we used $\partial p_{s}\left(\xi ,t\right)/\partial t=0$ from J=0. In Equation (45), non-zero of $ds^{p}\left(z_{t}\right)/dt$ indicates the time-dependence of $s^{p}\left(z\right)$, and the non-equilibrium states of the individual curve trajectories. Furthermore, $d\left(z_{t}∥\xi_{t}\right)\to 0$ indicates $p\left(z_{t}\right)\to p_{s}\left(\xi_{t}\right)$, which means the relaxation to a thermal equilibrium state, and $d\left(z_{t}∥\xi_{t}\right)\to const.\;\left(\neq 0\right)$ indicates the convergence to other (non-equilibrium) stationary states.

Taking the ensemble average with respect to $z_{t}$, the KL divergence between $z_{t}$ and $\xi_{t}$ is derived as:

(46) $$D\left(z∥\xi \right)=\langle d{\left(z_{t}∥\xi_{t}\right)\rangle }_{z_{t}}≃\langle \ln{\left|1-\frac{2t}{z_{t}{}^{2}}+O\left(\left|z_{t}{}^{-3}\right|\right)\right|}^{2}\rangle {}_{z_{t}},$$

From Equation (45), one can find that this quantity works as an indicator of the irreversibility and stationarity of the whole ensemble of the trajectories of the curves. We call $D\left(z∥\xi \right)$ (or $d\left(z_{t}∥\xi_{t}\right)$ depending on the situations) expressed by Equation (46) the *relative Loewner entropy*, which is used to evaluate the non-equilibrium state of the 2D trajectory on ℍ.

The relative Loewner entropy has a connection with phase space deformation under the conformal map $g_{t}$. If we take the time average of the non-additive part in Equation (43) after calculus, we obtain a form like the Lyapunov exponent [50] for the conformal map $g_{t}$,

(47) $$\lambda =\underset{T\to \infty }{\lim}\frac{1}{T}\overset{T}{\underset{t=0}{\sum }}\ln\left|\frac{dg_{t}\left(z_{t}\right)}{dz}\right|=\frac{1}{2}\bar{d\left(z_{t}∥\xi_{t}\right)},\;\;\;\;\;\;\left(T\gg 0\right).$$

Note that the overbar represents the time average. Here, the Lyapunov-type exponent λ defined by Equation (47) measures, rather than the sensitivity to the initial condition, the time-averaged phase space expansion (λ>0) and contraction (λ<0) of the neighborhood of the tip of the curve on ℍ under the map $g_{t}\left(z_{t}\right)$. Equations (45) and (47) show that it closely related to the total entropy production rate of $S^{p}\left(z\right)$. Note that an equilibrium state is characterized by λ=0 from Equation (47).

## 4. Numerical Tests

To realize the curves $\gamma_{\left[0,\;t\right]}$ of our model, numerical simulations were performed using the following methods. First, Langevin dynamics in Equation (4) was simulated by choosing the potential function as $V\left(\xi \right)=\left(1/2\right)a\xi^{2}$, such that $\alpha \left(\xi_{t}\right)=-a\xi_{t}$, where a is a positive constant. Consequently, the driving function can be described by a linear Langevin equation. The discretization of the Langevin equation is performed using a method similar to that in ref. [51], that is:

(48) $$\xi_{i}=\xi_{i-1}-a\tau \xi_{i-1}+\sqrt{\kappa \tau }W_{i-1},\;\;\left(i\geq 1\right).$$

Here, τ is a sufficiently small unit time interval (t=iτ), and $W_{i}$ is the white Gaussian noise with mean 0 and variance 1.0. κ is the diffusion parameter, which is the same as that in Equation (4). The initial condition is set as $\xi_{0}=\sqrt{\kappa /2a}$, which is the condition, derived from fluctuation dissipation theorem [52], that the driving function is in an equilibrium state from the initial state. After simulating Langevin dynamics using Equation (48), the shifted driving function $\xi_{i}-\xi_{0}$ was calculated such that its initial condition is zero. We note that this operation makes the curves start at the origin in the theoretical scheme; however, this is not necessary for our numerical computation algorithm described below, because we use the time differences of $\xi_{i}$ only. For the numerical realizations of the curves $\gamma_{\left[0,\;n\right]}$, we employed the zipper algorithm using the map derived from the vertical slit map [53,54], which is described as follows:

(49) $$\gamma_{\left[0,n\right]}=\left\{z_{0}\left(=0\right),z_{1}=f_{1}\left(0\right),z_{2}=f_{1}\circ f_{2}\left(0\right),\ldots ,z_{n}=f_{1}\circ \cdots \circ f_{n-1}\circ f_{n}\left(0\right)\right\},\;\;\;\;\;$$

where:

(50) $$f_{i}\left(z\right)=\Delta \xi_{i}+\sqrt{z^{2}-4\tau },\;\;\;\;\;\;\;\;\Delta \xi_{i}\equiv \xi_{i}-\xi_{i-1}.$$

**Figure 1:**
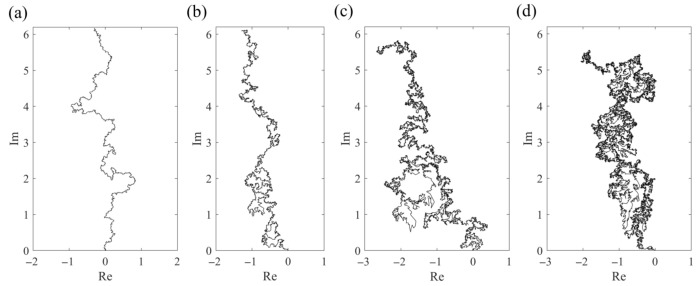
Typical examples of numerically realized curves $\gamma_{\left[0,\;n\right]}$ of a generalized SLE on upper half-plane ℍ. Simulations were performed with $n=1.0\times 10^{5}$ and $\tau =1.0\times 10^{-4}$. The drift term was chosen as a=1.5. (**a**) κ=2.0, (**b**) κ=4.0, (**c**) κ=6.0, and (**d**) κ=8.0.

Figure 1 shows the examples of the curves $\gamma_{\left[0,\;n\right]}$ calculated using the above algorithm ($n=1.0\times 10^{5}$ and $\tau =1.0\times 10^{-4}$). The drift term was chosen as a=1.5 (Figure 1a κ=2.0, Figure 1b κ=4.0, Figure 1c κ=6.0, and Figure 1d κ=8.0). It was observed that the phase of the curves varies depending on κ in a similar manner to that in the usual SLE [31] although the rigorous mathematical analysis is required to discuss this problem formally.

**Figure 2:**
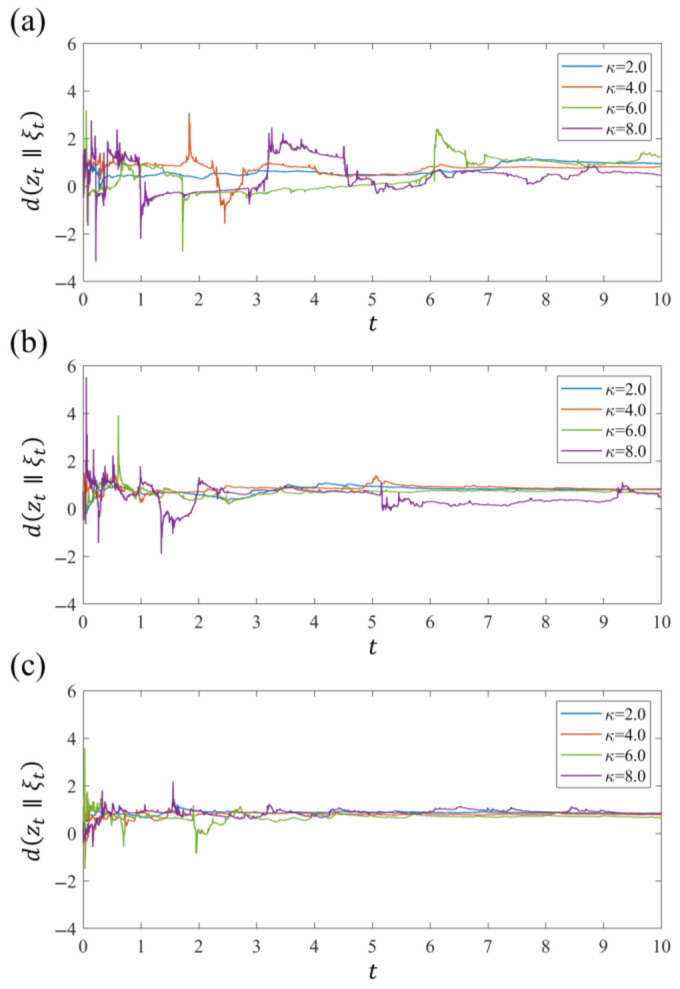
Temporal behaviors of relative Loewner entropy $d\left(z_{t}∥\xi_{t}\right)$ calculated as $\ln{\left|1-\frac{2t}{z_{t}{}^{2}}\right|}^{2}$. (**a**) a=0.5, (**b**) a=1.0, and (**c**) a=1.5. Each figure includes plots for κ=2.0, 4.0, 6.0, and 8.0. Note that we omitted the term $O\left(\left|z_{t}{}^{-3}\right|\right)$ in Equation (44), assuming the long-time behavior of $d\left(z_{t}∥\xi_{t}\right).$ The numerically estimated (maximum) absolute errors (A. E.) of this approximation exponentially decrease to A.E.≃0.2 in the range t≃1.5–2.0, and converge to A. E.≃0.08 in t≃5.0. After these time regions, the relation $\ln{\left|1-\frac{2t}{z_{t}{}^{2}}\right|}^{2}-A.E.<d\left(z_{t}∥\xi_{t}\right)<\ln{\left|1-\frac{2t}{z_{t}{}^{2}}\right|}^{2}+A.E.$ is guaranteed to be independent of a and κ.

First, the numerical experiments using the relative Loewner entropy are aimed towards verifying the non-stationary properties of the individual trajectories of the tip $z_{t}$ on ℍ calculated by the above procedures. Particularly, we estimate κ- and a-dependences to their dynamical regimes. Figure 2a–c shows the temporal behaviors of $d\left(z_{t}∥\xi_{t}\right)$, calculated by $\ln{\left|1-\frac{2t}{z_{t}{}^{2}}\right|}^{2}$, for a=0.5, 1.0, and 1.5, respectively. Each figure includes the plots for κ=2.0, 4.0, 6.0, and 8.0. For a=0.5, $d\left(z_{t}∥\xi_{t}\right)$ fluctuated violently, particularly for large κ, even after a long time passed (Figure 2a). For a=1.0, there were less violent fluctuations in $d\left(z_{t}∥\xi_{t}\right)$ than those for a=0.5, and they seemed to loosely converge to positive values except for κ=8.0 (Figure 2b). For a=1.5, the convergence of $d\left(z_{t}∥\xi_{t}\right)$ was more valid than that for a=1.0, indicating the non-equilibrium stationary state of the trajectory of $z_{t}$ (Figure 2c). These results indicate the tendency that smaller a and larger κ result in the non-stationary states of the trajectories.

**Figure 3:**
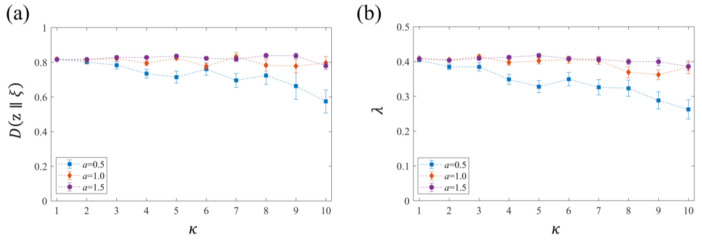
Parameter dependence of relative Loewner entropy $D\left(z∥\xi \right)$ and the Lyapunov-type exponent λ. (**a**) κ-dependence of $D\left(z∥\xi \right)$ at t=10.0 for a=0.5, 1.0, and 1.5. The ensemble average was taken over 50 realizations of $d\left(z_{t}∥\xi_{t}\right)$ and error bars show the standard error (S. E.). (**b**) κ-dependence of λ for a=0.5, 1.0, and 1.5. The time average in Equation (47) was taken over the range t=5.0–10.0, where the approximation of $d\left(z_{t}∥\xi_{t}\right)$ is most valid. The markers represent the mean value of λ calculated from 50 realizations and error bars show the S. E.

Subsequently, we estimated the non-equilibrium (irreversible) characteristic of the ensemble of the trajectories by calculating $D\left(z∥\xi \right)$ and λ. Figure 3a shows the κ-dependence of $D\left(z∥\xi \right)$ at t=10.0 for a=0.5, 1.0, and 1.5. The ensemble average was taken over 50 realizations of $d\left(z_{t}∥\xi_{t}\right)$. For a=0.5, the increase of κ results in the decrease in $D\left(z∥\xi \right)$, which indicates the loss of irreversibility. Contrarily, for a=1.0 and a=1.5, $D\left(z∥\xi \right)$ takes a relatively constant value independent of κ. This result suggests that the larger drift term in the driving function stabilizes $D\left(z∥\xi \right)$ regardless of the strength of the diffusion parameter κ. Figure 3b shows the κ-dependence of λ for a=0.5, 1.0, and 1.5. The time average in Equation (47) was taken over the range t=5.0–10.0, where the approximation of $d\left(z_{t}∥\xi_{t}\right)$ using $\ln{\left|1-\frac{2t}{z_{t}{}^{2}}\right|}^{2}$ is most valid. It was found that the behavior of the average values of λ has a similarity to that of $D\left(z∥\xi \right)$. The significant κ-dependence of λ was observed only for a=0.5, and λ tends to be a relatively constant value for a=1.0 and 1.5. Consequently, we found that the system with smaller a and larger κ tends to approach a possible equilibrium state (i.e., the driving function) at an ensemble level, although the trajectory-level stationarity becomes ambiguous. This result is related to the fractal dimension and phase of the curve. Furthermore, the similarity between the behavior of 2λ and that of $D\left(z∥\xi \right)$ implies the ergodicity of the curve trajectories, i.e., the existence of their (non-equilibrium) stationary distribution.

## 5. Discussion

We formulated non-equilibrium statistical mechanics of a generalized SLE using an information-thermodynamical approach. The SLE framework provides a unique information-theoretic scheme, in which irreversible non-equilibrium systems (i.e., the curves in the physical plane) are encoded into reversible equilibrium systems (i.e., the driving functions in the mathematical plane). We showed that this encoding operation is available due to the one-to-one correspondence between the curves and driving functions, and the phase space deformation of the conformal map $g_{t}$. The advantages of encoding a 2D non-equilibrium trajectory into reversible Langevin dynamics are summarized as follows.

1. The Jarzynski equality and the second law of thermodynamics were generalized in terms of information theory. Our result $0\leq \langle s_{i}^{p}-\ln R_{m}\rangle$ in Equation (41) is an extension of Seifert’s expression $0\leq \langle \Delta s_{tot}\rangle$ (see refs. [10,19]). Furthermore, the term $\ln R_{m}$ can also be interpreted as the feedback information term, denoted as I in ref. [23], in Sagawa’s information thermodynamics. Hence, incorporating the relaxation process of an equilibrium state into the theory of the non-equilibrium dynamics enables us to extend the existing thermodynamical laws in an information-theoretic sense. This means that for an arbitrary 2D trajectory on ℍ in our model, the validity of the second law in the usual sense $0\leq \langle s_{i}^{p}\rangle$ is supported by the complete time reversibility of the corresponding driving function, and otherwise (i.e., if the driving function includes several irreversible characters), we must reuptake the generalized second law $0\leq \langle s_{i}^{p}-\ln R_{m}\rangle$.
2. The entropy describing the non-equilibrium states of the individual trajectories is decomposed into additive and non-additive parts. This provides us with a novel non-equilibrium entropic measure, which we refer to as the relative Loewner entropy. In the sense that the non-equilibrium ensemble is decomposed into an equilibrium ensemble and a certain function, our result in Equation (43) is analogous to the result of Penrose et al. [55].
3. If the driving function is in an equilibrium state, the relative Loewner entropy is used to determine the non-equilibrium properties (i.e., non-stationarity and changing rate of Gibbs entropy) of the 2D trajectories in the physical plane. This quantity indicates the phase space deformation under the conformal map $g_{t}$, and is closely related to the Lyapunov-type exponent.

These advantages suggest that non-equilibrium states are well understood when we assume the associated processes in an equilibrium in the theoretical framework. The equilibrium driving function works as an idealized thermal state, which is one of the stationary states that the curves potentially reach in the long-time limit. If the entropy of the curve trajectory completely coincides with that of driving function, in which the KL divergences become zero, the thermal equilibrium of the physical and mathematical planes can be equally characterized.

Then, we encounter the problem of providing the information-theoretic and physical meanings of the driving function. In the information-theoretic sense, the driving function is interpreted as an information source manipulating the 2D curve trajectories, which has full information about their equilibrium states. In this view, from the results of this study, we can conclude that:

1. If the entropy (information) of the driving function is completely communicated to the physical plane, the 2D trajectories are in the equilibrium states.
2. The non-equilibrium property of the trajectories is induced by the incomplete communication of the entropy (information) between the physical and mathematical planes.
3. The driving function can work as Maxwell’s Demon in the sense that it can control the feedback information $\ln R_{m}$.

These statements will make better sense when the integration of the information theory and thermodynamics is successfully undertaken. In addition, in a thermodynamical sense, the entropy of the driving function is interpreted as a conserved variable of the thermodynamic potential for the 2D non-equilibrium trajectories. Although non-equilibrium systems typically lack the conserved quantities, such as the Hamiltonian in the equilibrium physics, our result indicates that the entropy (or associated energy function) of the driving function is conserved, even if the corresponding curve retains the non-equilibrium state. A similar type of perspective was suggested in the mathematical context [56,57], where the invariance of the Dirichlet energy of the Loewner driving function (referred to as the *Loewner energy*) was demonstrated. For these reasons, we suggest that entropy of the Loewner driving function may be referred to as the *Loewner entropy*.

Most importantly, the two thermodynamically different systems in our model are linked via a family of conformal maps $g_{t}$, determined by the Loewner equation, and mathematically convertible. Hence, in a generalized SLE framework, the microscopically irreversible process can arise from a reversible (but time-dependent) transformation to the microscopically reversible process. This means that the Boltzmann paradox [58] in our model is caused by the small deviation between the two entropies (as shown in Equation (43)) induced by conformal transformations. In this regard, the relative Loewner entropy explicitly represents an exact difference between non-equilibrium and equilibrium states; therefore, it can be considered to be a candidate of a non-equilibrium entropy.

The growth processes of the SLE-type curve have been investigated as a problem of Laplacian random walk (LRW) [34,59], which is applied to the dielectric breakdown and polymer growth model, etc. [60,61]. By comparing the SLE processes with these physical models, it can be also found that the capacity t in the conformal map $g_{t}\left(z\right)$ is analogous to the usual physical time in the present model [54]. However, the time t in the Loewner evolution, referred to as the *Loewner time,* often has specific randomized time increments, which are obtained when we calculate the driving functions of arbitrary 2D trajectories [54]. In ref. [25], a similar type of random time was introduced to demonstrate the generic properties of entropy production as the *entropic time*, which enables mapping the entropy production in the non-equilibrium steady states into drift-diffusion processes. This result has a connection with the present results in the sense that the time-dependent transformation is implemented for the purpose of investigating non-equilibrium systems.

To apply our formulations, including the suggested irreversibility measure, to other various 2D self-organization phenomena (e.g., Ising systems, percolation models, turbulence, or biological/chemical morphologies), we must extend the descriptive ability of the SLE framework. In the present model, we chose the driving function as Langevin dynamics with a (linear or nonlinear) drift term. This is an example of a generalization of the SLE; however, numerous other possibilities remain for the extension of the SLE framework (e.g., combining with chaotic dynamical systems [32,37,62], *q*-deformation [63]). Therefore, future research following this study will clarify how our theoretical concepts aid the understanding of real self-organization phenomena, by considering appropriate approaches to the generalization of the SLE. We note that our formulations are still limited to the estimation of the entropic behavior of the non-equilibrium systems, and revealing other physical properties from the 2D trajectories requires further individual investigation of real systems, including experimental studies.

## 6. Conclusions

We formulated the generalized SLE driven by Langevin dynamics in the equilibrium state from the context of non-equilibrium statistical mechanics. The entropy production of the curve trajectories in the physical plane assumed a form of irreversible non-equilibrium systems, whereas the driving function was prepared in the reversible equilibrium system. We derived alternative types of the Jarzynski equality and the second law of thermodynamics by information-theoretic quantities. Furthermore, we showed, from the phase deformation ratio of the conformal maps, that the entropy of the curve can be decomposed into additive and non-additive parts. The non-additive part was numerically examined to estimate non-equilibrium properties of the system, and we refer to it as the relative Loewner entropy. These results suggest a novel perspective for non-equilibrium statistical physics to answer the question concerning the definition of a non-equilibrium entropy and the mechanisms of irreversibility.

## Data Availability

The data presented in this study are available on request from the corresponding author.
